# Dr. Isaac Ott

**Published:** 1916-02

**Authors:** 


**Dr. Isaac Ott,** University of Penn., 1869, died at his home in Easton, Pa., Jan. 1, aged 68. He was the author of a work on Internal Secretions and on Human Physiology.

